# Supplementary material for: Insights into intestinal barrier disruption during long-term gut Chlamydia colonization in mice: a single-cell transcriptomic approach
Source: Front Cell Infect Microbiol. 2025 Jul 25;15:1614009. doi: 10.3389/fcimb.2025.1614009 (PMC12331646; doi:10.3389/fcimb.2025.1614009)
Supplement: Supplementary file 1 [file DataSheet1.pdf]

Supplementary Material

1 Supplementary Figure Legends

Figure S1. ScRNA-seq reveals *Chlamydia* infection associated shift in intestinal cell composition. [A] Dot plot showing expression of representative markers in each cell type. [B] UMAP plot shows the distribution of different cell types. [C] Pie chart shows the cell number and proportion of each cell type. [D] Histogram shows the proportion of cell populations of each cell type in each sample.

Figure S2. ScRNA-seq reveals intestinal epithelial cell type-specific transcriptional response evoked by *Chlamydia*l infection. [A] Volcano plot shows DEGs results of different epithelial cell populations between *Chlamydia* vs Control groups. [B] Volcano plot shows DEGs results of different epithelial cell populations between De_*Chlamydia* vs Control groups.

Figure S3. Dysregulation of transcriptional regulatory networks plays an important role in defense against *Chlamydia* infection. [A] UMAP plot shows the AUC activity distribution of selected regulons in epithelial cells. [B] GO biological process results of target genes regulated by TFs specific to different epithelial cells. [C] Bar graphs show the number of differentially expressed TFs compared between different groups. [D] UMAP plot shows the AUC activity of Creb3(+) in each group. [E] The most enriched KEGG pathways of target DEGs regulated by Creb3 in GC. [F] Correlation scatter plot shows the interplay between Creb3 and Ido1 expression in GC.

Figure S4. Alteration of cell-cell interactions during *Chlamydia* infection. [A] Interactions of GC cells in different groups. [B] CEACAM signal pathway network in different groups. [C] OCLN signal pathway network in different groups. [D] Correlation scatter plot shows the interplay between Creb3 and P4hb expression in GC.

Fig.S5 Effects of *Chlamydia* infection on intestinal epithelium cell fate. [A] GO enrichment results of different module genes. [B] The pseudotime expression changes of Creb3 in different lineages and its expression differences among different groups in lineage 1.
