# Supplementary figures and images for: Insights into intestinal barrier disruption during long-term gut Chlamydia colonization in mice: a single-cell transcriptomic approach
Source: Front Cell Infect Microbiol. 2025 Jul 25;15:1614009. doi: 10.3389/fcimb.2025.1614009 (PMC12331646; doi:10.3389/fcimb.2025.1614009)

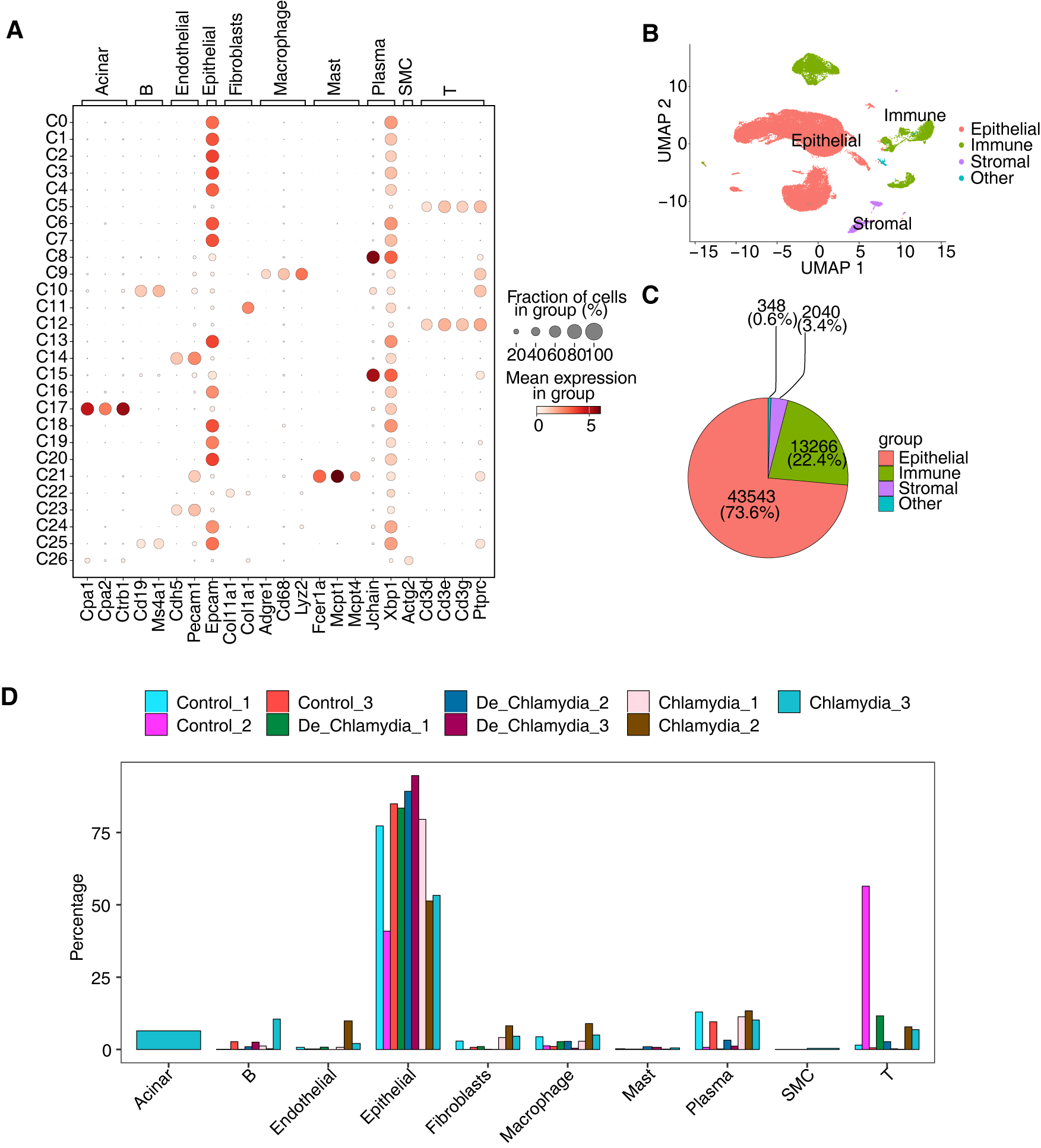

Supplement: Supplementary Figure 1 — ScRNA-seq reveals Chlamydia infection associated shift in intestinal cell composition. (A) Dot plot showing expression of representative markers in each cell type. (B) UMAP plot shows the distribution of different cell types. (C) Pie chart shows the cell number and proportion of each cell type. (D) Histogram shows the proportion of cell populations of each cell type in each sample. [file Image1.tif]

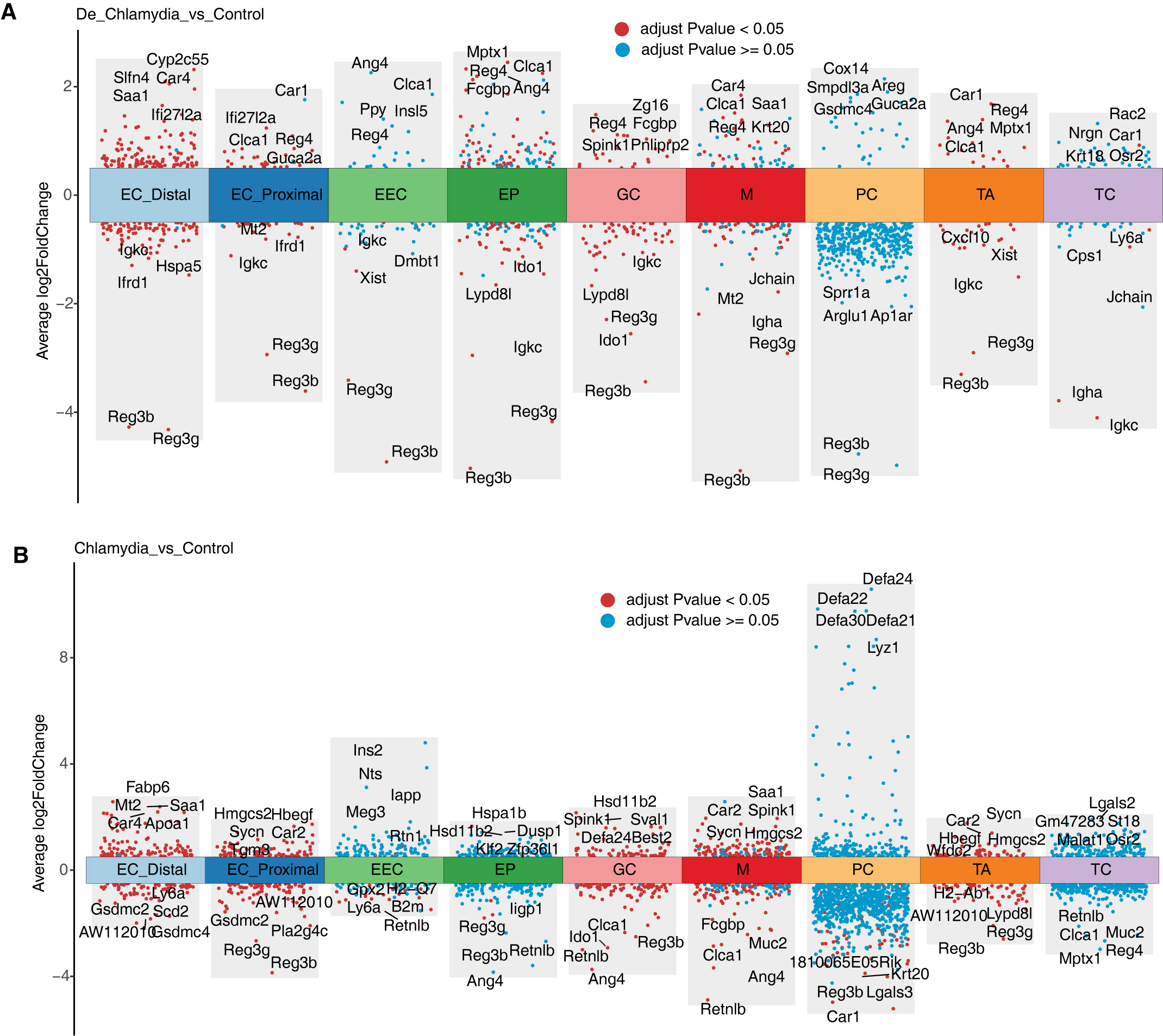

Supplement: Supplementary Figure 2 — ScRNA-seq reveals intestinal epithelial cell type-specific transcriptional response evoked by Chlamydial infection. (A) Volcano plot shows DEGs results of different epithelial cell populations between Chlamydia vs Control groups. (B) Volcano plot shows DEGs results of different epithelial cell populations between De_Chlamydia vs Control groups. [file Image2.tif]

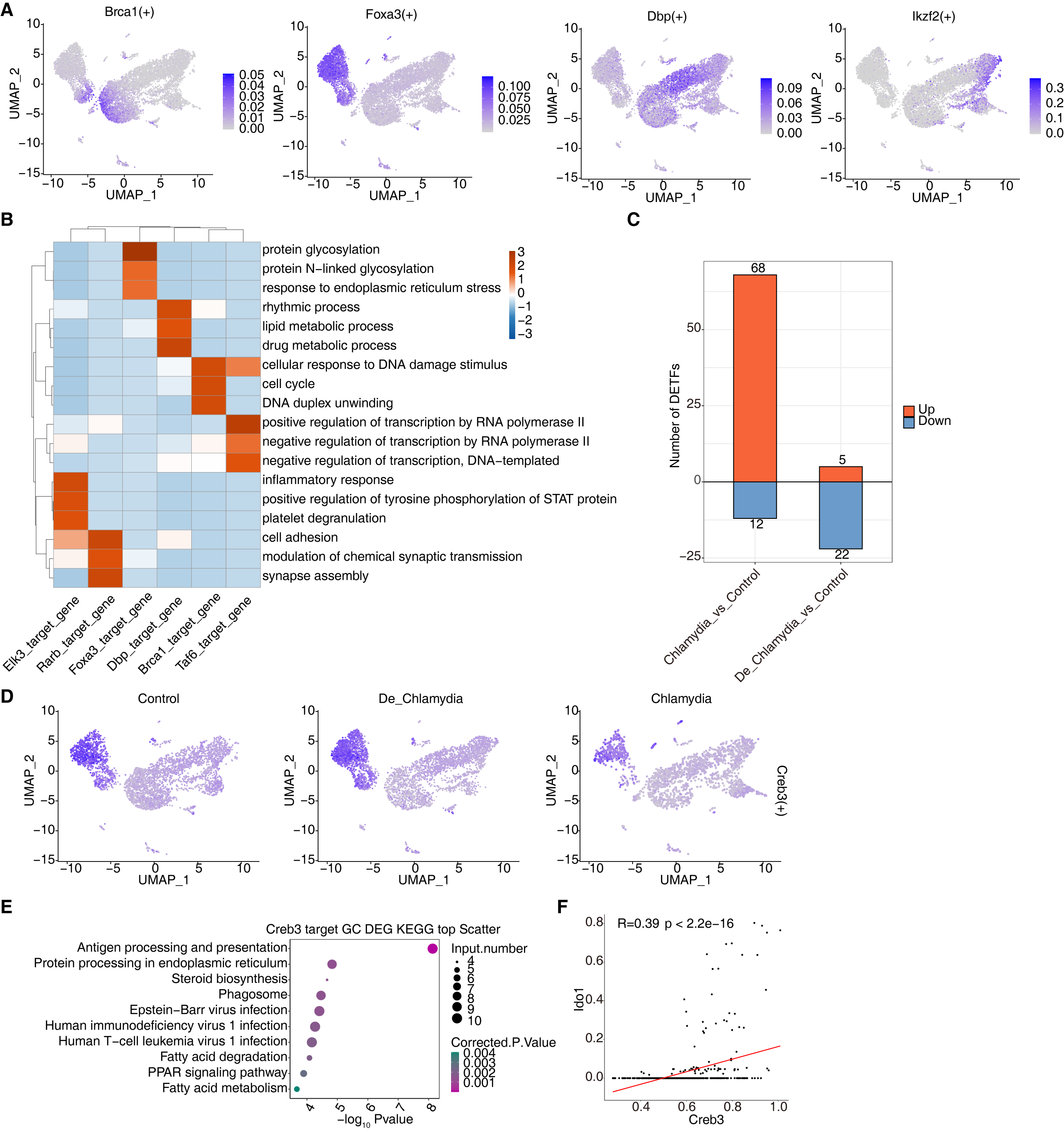

Supplement: Supplementary Figure 3 — Dysregulation of transcriptional regulatory networks plays an important role in defense against Chlamydia infection. (A) UMAP plot shows the AUC activity distribution of selected regulons in epithelial cells. (B) GO biological process results of target genes regulated by TFs specific to different epithelial cells. (C) Bar graphs show the number of differentially expressed TFs compared between different groups. (D) UMAP plot shows the AUC activity of Creb3(+) in each group. (E) The most enriched KEGG pathways of target DEGs regulated by Creb3 in GC. [F] Correlation scatter plot shows the interplay between Creb3 and Ido1 expression in GC. [file Image3.tif]

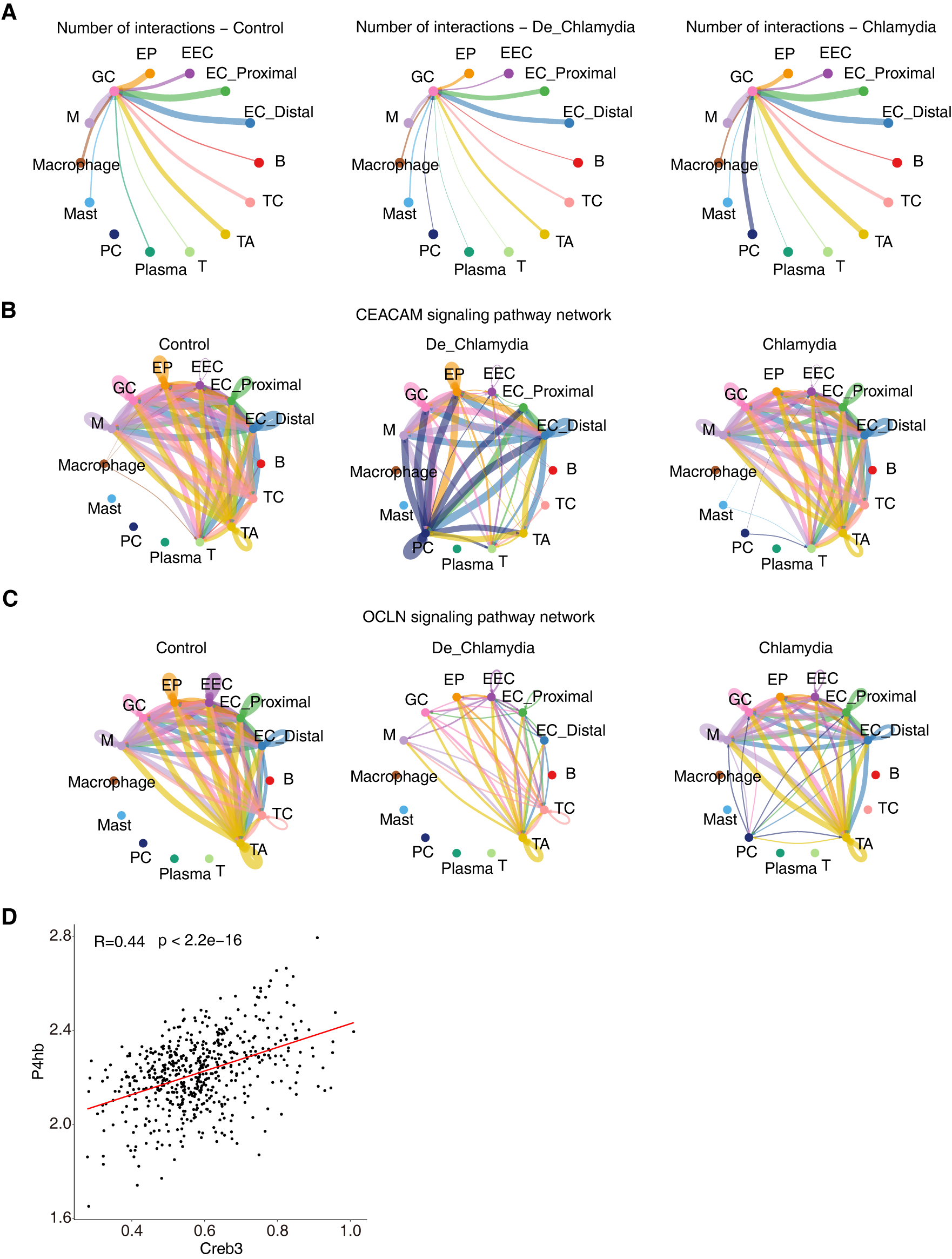

Supplement: Supplementary Figure 4 — Alteration of cell-cell interactions during Chlamydia infection. (A) Interactions of GC cells in different groups. (B) CEACAM signal pathway network in different groups. (C) OCLN signal pathway network in different groups. (D) Correlation scatter plot shows the interplay between Creb3 and P4hb expression in GC. [file Image4.tif]

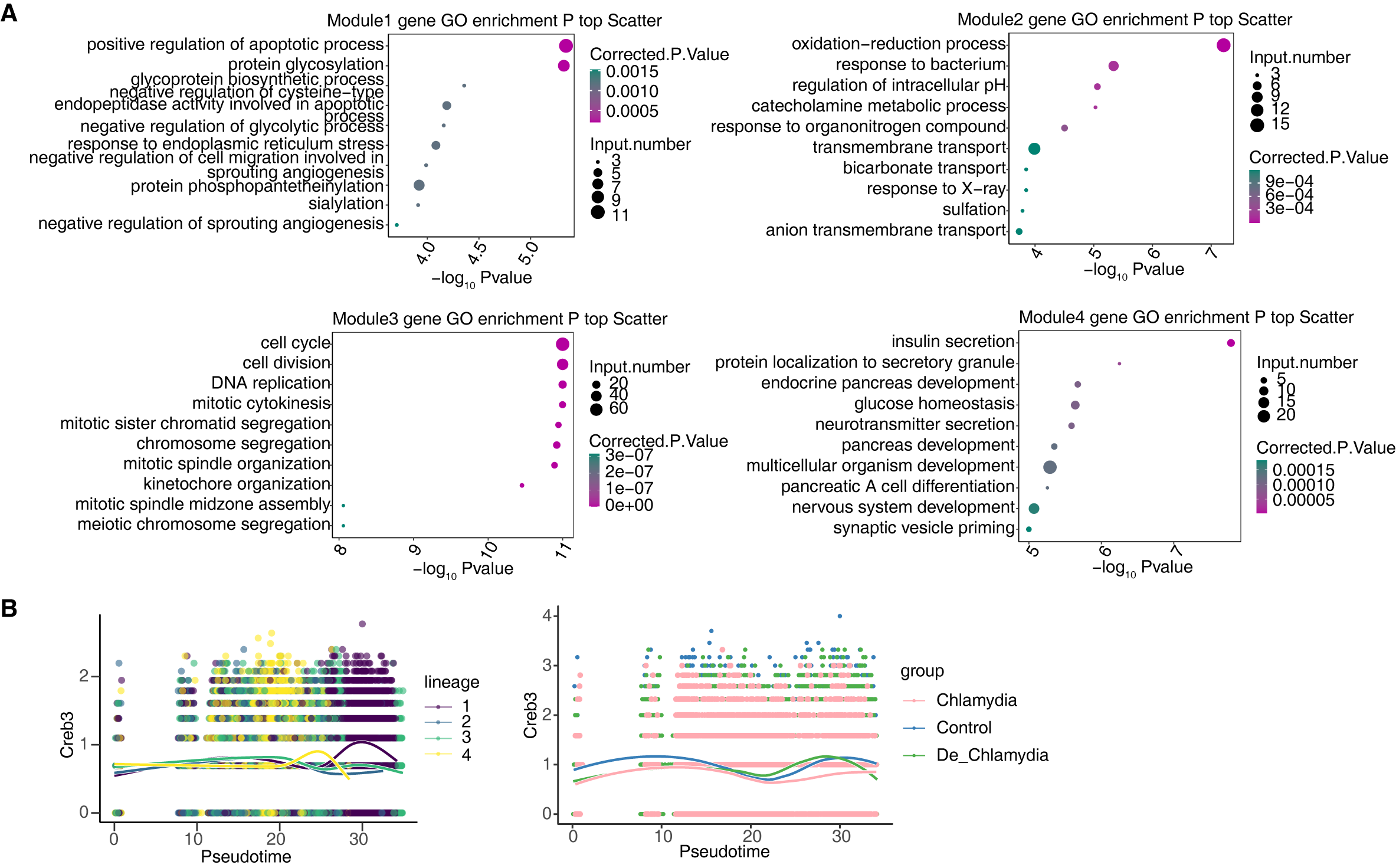

Supplement: Supplementary Figure 5 — Effects of Chlamydia infection on intestinal epithelium cell fate. (A) GO enrichment results of different module genes. (B) The pseudotime expression changes of Creb3 in different lineages and its expression differences among different groups in lineage 1. [file Image5.tif]

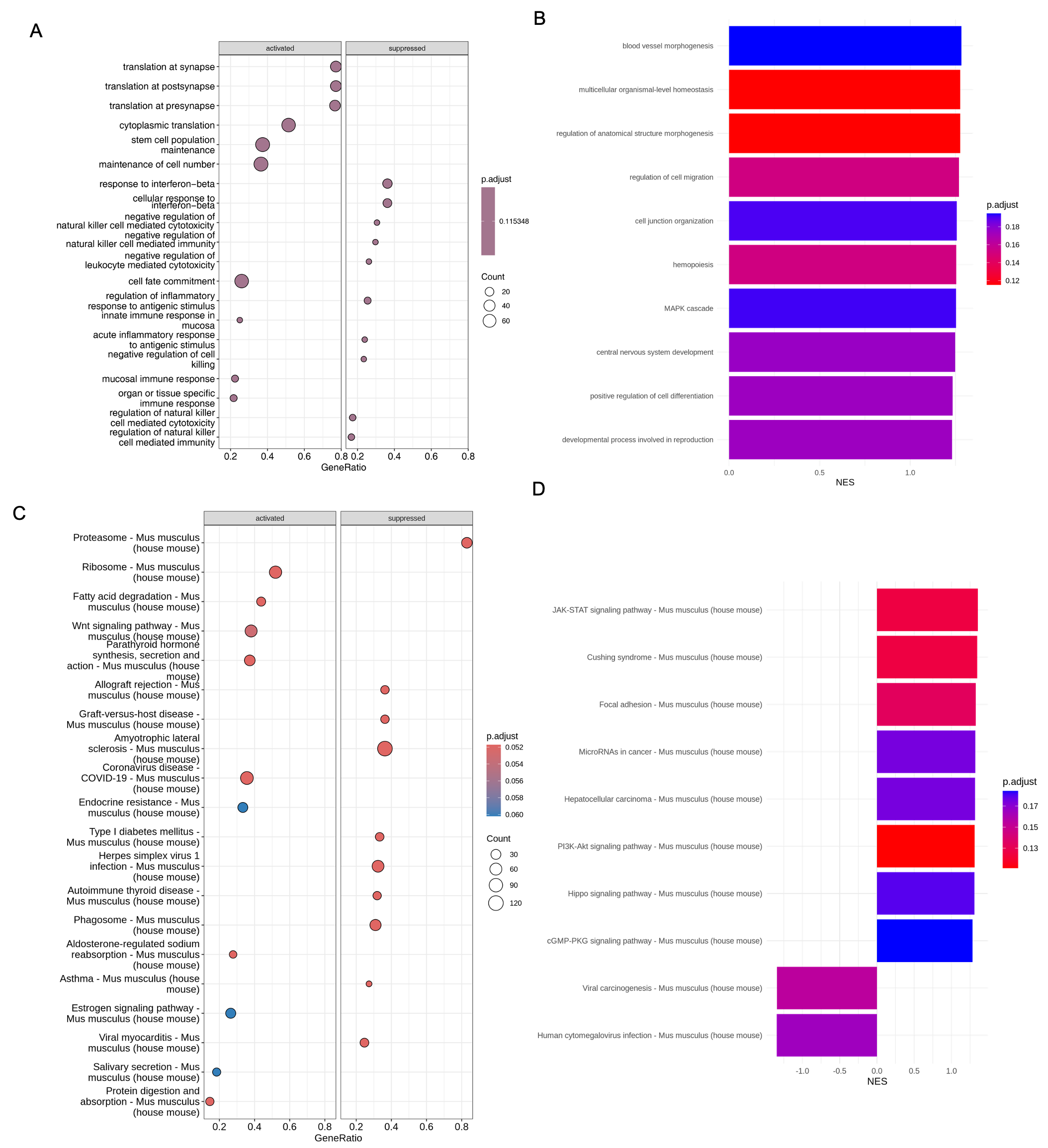

Supplement: Supplementary Figure 6 — GSEA Analysis Panels (A, C): The top 10 significant pathways identified through Gene Set Enrichment Analysis (GSEA) are displayed, with a p-value threshold of <0.05. The depth of color indicates the level of significance, with darker shades representing more significant pathways. The size of the dots corresponds to the number of genes involved in each pathway. The x-axis represents the generation value of the pathways. Panels (B, D): These panels present the results of all pathways ranked based on the top 10 significant adjusted p-values (p.adjust). Pathways with a normalized enrichment score (NES) less than 0 indicate inhibition (suppressive pathways), while NES greater than 0 signifies activation (activating pathways). The depth of color again reflects the level of significance, and the x-axis displays the NES values. [file Image6.png]
